# Regulation-Structured Dynamic Metabolic Model Provides a Potential Mechanism for Delayed Enzyme Response in Denitrification Process

**DOI:** 10.3389/fmicb.2017.01866

**Published:** 2017-09-29

**Authors:** Hyun-Seob Song, Dennis G. Thomas, James C. Stegen, Minjing Li, Chongxuan Liu, Xuehang Song, Xingyuan Chen, Jim K. Fredrickson, John M. Zachara, Timothy D. Scheibe

**Affiliations:** Pacific Northwest National Laboratory, Richland, WA, United States

**Keywords:** nitrogen cycle, denitrification, hyporheic zone, environmental microbial community, trait-based modeling, the cybernetic approach, functional enzyme-based modeling

## Abstract

In a recent study of denitrification dynamics in hyporheic zone sediments, we observed a significant time lag (up to several days) in enzymatic response to the changes in substrate concentration. To explore an underlying mechanism and understand the interactive dynamics between enzymes and nutrients, we developed a trait-based model that associates a community's traits with functional enzymes, instead of typically used species guilds (or functional guilds). This enzyme-based formulation allows to collectively describe biogeochemical functions of microbial communities without directly parameterizing the dynamics of species guilds, therefore being scalable to complex communities. As a key component of modeling, we accounted for microbial regulation occurring through transcriptional and translational processes, the dynamics of which was parameterized based on the temporal profiles of enzyme concentrations measured using a new signature peptide-based method. The simulation results using the resulting model showed several days of a time lag in enzymatic responses as observed in experiments. Further, the model showed that the delayed enzymatic reactions could be primarily controlled by transcriptional responses and that the dynamics of transcripts and enzymes are closely correlated. The developed model can serve as a useful tool for predicting biogeochemical processes in natural environments, either independently or through integration with hydrologic flow simulators.

## Introduction

Microbes in terrestrial and aquatic ecosystems are primary drivers of biogeochemical processes, including carbon and nitrogen cycling (Gougoulias et al., 2014). Predicting ecosystem functions and dynamics in a varying environment therefore requires a mechanistic understanding of how microorganisms interact with each other to catalyze biogeochemical functions. Microbial communities are complex adaptive systems; their dynamics and emergent properties are difficult to understand and predict (Konopka et al., 2015). A long time lag in the microbial responses to changes in local environment is an example of complex community dynamics commonly observed in the field and experimental studies, but the underlying reasons are poorly understood (Wood et al., 1995; Shade et al., 2012). Metabolic time lags in microbes can affect the fate and transport of microbial substrates, particularly when the timescales of both metabolic lag and transport are comparable (Nilsen et al., 2012). Currently, there are no fully mechanistic approaches for incorporating metabolic lags into reactive transport models. Existing models account for metabolic lags by using temporal convolution integrals (Nilsen et al., 2012) or by introducing exposure time as an additional dimension (or coordinate; Wood et al., 1995). These models, however, do not address the fundamental mechanisms that lead to such delayed metabolic responses; thus, limiting their use for prediction.

In a recent experimental study (Li et al., 2017), we reported the complex dynamics of microbial communities that drive denitrification in hyporheic zone sediments from the Hanford Reach of the Columbia River in Washington state. The hyporheic zone is a biologically active domain where (nitrogen-limited, but carbon-abundant) surface water and (carbon-poor, but nitrogen-enriched) groundwater mix (Stegen et al., 2016). Denitrification in hyporheic zone sediments is mediated by microbial communities through multiple stages of reduction reactions from ${\text{NO}}_{3}^{-}$ to N_2_, with each step catalyzed by distinct enzymes. Our experimental data showed a significant time lag in in the synthesis of enzymes that catalyze ${\text{NO}}_{3}^{-}$ reduction to ${\text{NO}}_{2}^{-}$. Microbial communities continued to express catalyzing enzymes even after ${\text{NO}}_{3}^{-}$ was depleted. Enzyme concentrations did start to decrease only several days after ${\text{NO}}_{3}^{-}$ reduction was completed and were maintained at base levels thereafter.

Understanding the processes governing delayed enzyme synthesis and catalysis and its translation into quantitative modeling are essential for predicting biogeochemical dynamics, particularly in dynamic environments such as the hyporheic zone where chemical conditions can change rapidly in response to hydrologic exchange. For these purposes, we developed a new concept of modeling that accounts for the regulated synthesis of enzymes through transcriptional and translational processes. The developed model collectively describes biogeochemical dynamics in terms of functional enzymes synthesized in a community, without directly considering the dynamics of individual species or their guilds that synthesize those enzymes. Using this model, we explored how the dynamic interplay between regulatory molecules such as transcripts, enzymes, and internal resource molecules affects biogeochemical functions. The resulting model not only provided an excellent fit to data (both nutrient and enzyme dynamics), but also suggested a complex regulatory machinery in microbes as a plausible mechanism for the observed time lags in enzyme responses. The model also provided novel insights into the biological roles of transcripts as a control point of dynamic metabolic regulation.

The microbial community model developed in this article provides several new conceptual components for microbial community modeling, which we summarize in the following section as an essential pre-requisite for understanding the idea of the proposed model framework and the implications of the simulation results.

## Background

### Functional enzyme-based formulation

Modeling environmental microbial communities is challenging, primarily due to immense complexity in their compositional diversity (Song et al., 2014). A common idea to alleviate this structural complexity is trait-based modeling, which maps organism-based information into a functional space (Allison, 2012; Boon et al., 2014). The trait-based modeling often considers grouping organisms that share certain metabolic functional similarities (i.e., traits) into a fewer number of species guilds or functional guilds (Taffs et al., 2009; Jin and Roden, 2011; Bouskill et al., 2012). This guild-based grouping can be less effective, however, in the case where microorganisms can perform multiple functions (functional versatility) that are partially overlapping with one another (functional degeneracy) in a dynamically changing environment (Whitacre and Bender, 2010; Song et al., 2015). As a consequence, the number of functional guilds (and accordingly, the number of parameters) can become significantly large.

As an alternative, the complexity of microbial communities can be reduced by associating a community's metabolic traits with *functional enzymes*. This enzyme-based approach views a microbial community as a collective assembly of metabolic capabilities as opposed to a complex network of individual organisms or their groups (e.g., functional guilds), therefore requiring no *a priori* knowledge of the functional roles of individual organisms.

**Figure 1:**
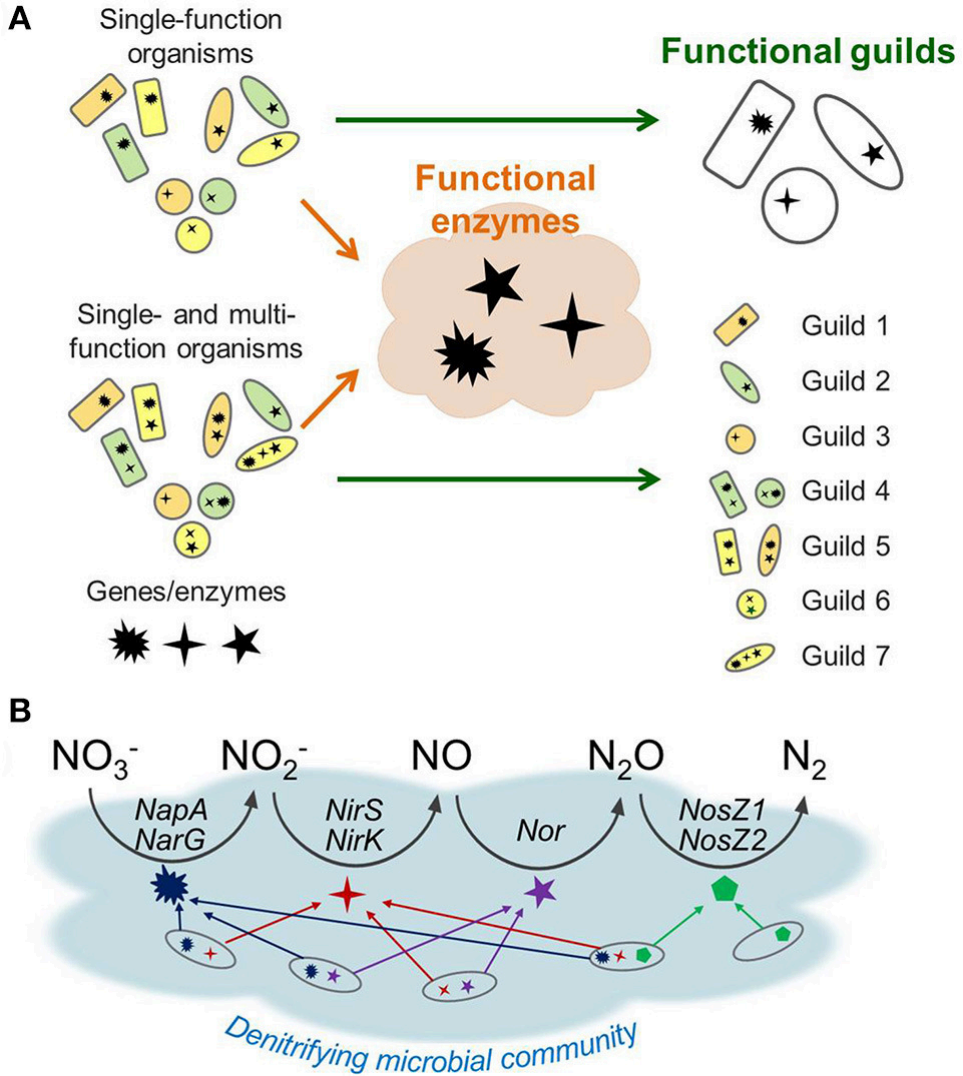
A schematic representation of trait-based microbial community modeling strategies: **(A)** functional guild- vs. functional enzyme-based formulations, **(B)** enzyme-based modeling of denitrification. In **(A)**, communities are considered as being composed of specialists (that can express one functional enzyme) only, or being mixed with specialists and functionally versatile organisms (that can express multiple enzymes). In **(B)**, the latter case is illustrated using denitrification, which is composed of four steps of reduction catalyzed by distinct sets of genes and enzymes: NapA and NarG for ${\text{NO}}_{3}^{-}$ reduction to ${\text{NO}}_{2}^{-}$, NirS and NirK for ${\text{NO}}_{2}^{-}$ to NO, Nor for NO to N_2_O, and NosZ1 and NosZ2 for N_2_O to N_2_.

Figure 1 illustrates how the enzyme- and guild-based formulations can be different, particularly with respect to model structure and parameterization. Figure 1A shows two cases where a microbial community is composed of (1) single-function organisms only (top) or (2) a combination of single- and multi-function organisms (bottom). These functions denote the microbes' capability of expressing enzymes that catalyze specific biogeochemical reactions such as those in denitrification. Therefore, single-function organisms (specialists) express one functional enzyme, while multi-function organisms (generalists) express multiple enzymes. The functional guild-based approach groups organisms based on the type and number of metabolic functions each guild catalyzes. For the community composed of specialists only, this approach can successfully reduce nine species into three guilds that express the same enzymes. In this case, the enzyme-based formulation is not much different from the species guild-based approach. If the community contains member organisms expressing multiple functional enzymes, the guild-based reduction results in the expanded number of guilds, implying the increase in the number of model parameters. In contrast, the enzyme-based approach reduces the model complexity by focusing on the functional enzymes independent of associated guilds. As a result, the functional enzyme-based approach only requires the identification of the dynamics of three enzyme classes regardless of the metabolic characteristics of organisms. Due to its focus on enzyme pool, the enzyme-based model carries no information on whether a given function is occurring within one or multiple organisms.

Figure 1B provides a schematic of the functional enzyme-based approach for denitrification, which is composed of four catalytic steps that reduce ${\text{NO}}_{3}^{-}$ to N_2_. The functionally versatile organisms (generalists) can contribute to more than one catalytic step as implied by the multiple arrows. Since the enzyme functions are directly modeled, the enzyme-based formulation does not have to track which organisms are involved in each step; thereby reducing the number of parameters to describe the community dynamics in comparison to the guild-based formulation.

### The cybernetic description of metabolic regulation

The enzyme-based formulation describes an entire microbial community as a single unit that responds to the environment by regulating its metabolism through expression of genes and enzymes. As a key advantage, this approach facilitates the application of existing dynamic modeling platforms that have been developed to predict metabolic behaviors of single organisms (Song et al., 2014; Vasilakou et al., 2016). In this regard, the cybernetic approach developed by Ramkrishna and co-workers (Ramkrishna, 1983; Ramkrishna and Song, 2012; Young, 2015) provides an ideal platform for the enzyme-based microbial community modeling due to its rational description of metabolic regulation through the control of enzyme syntheses and activities.

The cybernetic model postulates organisms as teleonomic systems that regulate metabolism toward achieving a certain metabolic objective (e.g., maximization of nutrient uptake rate or growth rate). This postulate removes the need to account for detailed mechanistic information on regulation, which is generally unknown for environmental organisms. The term “cybernetics” denotes a goal-seeking aspect of metabolism (Wiener, 1961; Mayr, 1988). The predictive power of cybernetic approach has been successfully demonstrated over the past few decades through a variety of challenging case studies of modeling metabolic switches in varying environments (Dhurjati et al., 1985; Kompala et al., 1986; Turner et al., 1988; Alexander and Ramkrishna, 1991; Baloo and Ramkrishna, 1991; Straight and Ramkrishna, 1991; Varner, 2000; Kim et al., 2008; Young et al., 2008; Song et al., 2009, 2013b; Song and Ramkrishna, 2010, 2011; Franz et al., 2011), dynamic response to genetic perturbations (Young et al., 2008; Song and Ramkrishna, 2012), and nonlinear behavior such as multiple metabolic states (Namjoshi et al., 2003; Kim et al., 2012; Song and Ramkrishna, 2013).

### Accounting for the dynamic interplay among regulatory molecules

**Figure 2:**
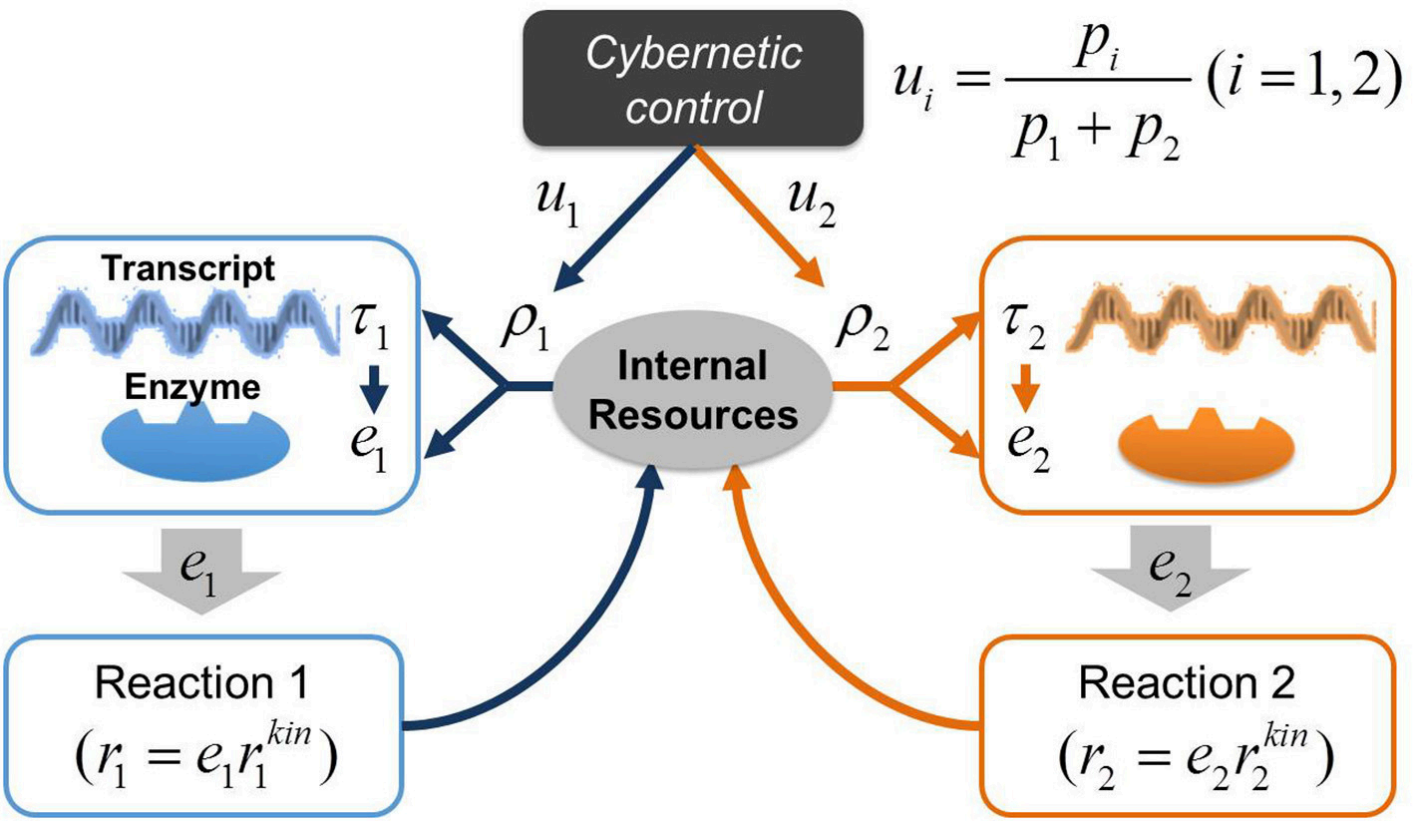
A schematic representation of the control structure in the RSCM. The variable *u*_*i*_ controls the synthesis of resource (ρ_*i*_) based on the contribution of individual reactions to the postulated metabolic objective (i.e., return-on-investment *p*_*i*_, e.g., *p*_*i*_ = *r*_*i*_); the synthesis of transcript (τ_*i*_) is subsequently determined by ρ_*i*_; the synthesis of *e*_*i*_ is determined by both τ_*i*_ and ρ_*i*_; the enzyme *e*_*i*_, in turn, catalyzes reaction (*r*_*i*_).

In a previous study, we applied the cybernetic approach to model the dynamics of denitrification process driven by *single* organisms (Song and Liu, 2015). The focus of this previous work was to simulate sequential utilization of multiple electron acceptors associated with denitrification and did not include enzyme measurements for model parameterization and validation. Due to the relatively simple description of the regulatory process, this form of cybernetic model is inappropriate for predicting a long enzymatic lag we observed in a denitrifying microbial community. Therefore, in this work, we significantly extended the previous model by elaborating cellular regulation program by accounting for the dynamic interplay among transcripts, enzymes, and internal resources. Following the central dogma of molecular biology (Crick, 1970), we modeled the regulation process in microbes based on transcription and translation: (1) the synthesis of transcripts from DNA and other internal resource molecules (such as ATP, NADH, DNA polymerases, RNA polymerases, ribosomes, etc.), and (2) the subsequent synthesis of enzymes from transcripts and internal resources. Note that the coupling between transcripts and enzymes is not occurring in a linear fashion due to the involvement of internal resources in both transcription and translation. We have termed this approach the Regulation-Structured Cybernetic Model (RSCM). Figure 2 illustrates the control structure of the RSCM for two competing reactions catalyzed by distinct enzymes.

The idea of elaborating the cybernetic regulation model at the transcriptional and translational levels was also considered previously in the literature (Varner, 2000), the regulation structure of which is, however, much more complex than the RSCM due to the direct consideration of the control of inductive synthesis of both transcripts and enzymes. In contrast, the RSCM indirectly realizes those controls only at the resource generation stage (see Figure 2 and model equations in the next section). As another key distinction, the RSCM accounts for the dynamics of internal resources and their dynamic interplay with transcripts and enzymes, which was neglected in the previous work.

## Methods

### Experimental data

We structured and parameterized the denitrification model based on the experimental data collected by the Pacific Northwest National Laboratory (PNNL)'s subsurface biogeochemistry research group. As all experimental details on data collection and enzyme assay are published elsewhere (Li et al., 2017), we here provide only a brief summary. Batch denitrification experiments under anaerobic conditions were performed using the Columbia River hyporheic zone sediments collected from the Hanford Reach. These sediment samples were mixed with synthetic groundwater. Experimental data measured for model parameterization include the concentrations of gas phase nitrous oxide (N_2_O), dissolved nitrate (${\text{NO}}_{3}^{-}$), nitrite (${\text{NO}}_{2}^{-}$), acetate, dissolved inorganic carbon (DIC), and functional enzymes. Concentrations of functional enzymes were quantified using the PNNL-developed signature peptide-based method. The targeted quantification of functional enzymes include dissimilatory ${\text{NO}}_{3}^{-}$ reductases (NapA and NarG) and dissimilatory N_2_O reductase (NosZ), which represent the first and last steps in denitrification. Other enzymes associated with intermediate steps (such as NirS/NirK and Nor) were not measured. Nutrient concentrations were determined by analyzing three replicates. The enzyme (NarG, NapA, NosZ1, and NosZ2) concentrations were obtained as the average of their two signature peptide concentrations: each signature peptide was determined from two replicates. Dissimilatory nitrate reduction to ammonium (DNRA) is another potential reaction pathway for the anaerobic reduction of nitrate, but the experimental data by Li et al. (2017) provide several evidences indicating that denitrification is a major reaction pathway active in their experiments. First of all, the ammonia assay detected no formation of ammonium, which is the end product of DNRA. From the same samples, however, the formation of N_2_O (an intermediate during denitrification) was detected. While it is known that N_2_O could also be released as a byproduct of DNRA (Stevens et al., 1998; Kraft et al., 2011), this could also be an evidence with no ammonium detected. Finally, Li et al. measured NosZ1 and NosZ1 enzymes that catalyze the reduction of N_2_O to N_2_, the final reduction step in denitrification. The temporal profiles of NosZ1/NosZ2 enzyme concentrations were qualitatively similar to those of NarG/NapA enzymes, indicating their coupling as the first and last steps of denitrification.

### Denitrification reaction network and stoichiometry

Based on the experimental data by Li et al. (2017) described above, we developed a biogeochemical model with a focus on denitrification. Denitrification is an anaerobic process in which ${\text{NO}}_{3}^{-}$ is reduced to N_2_ gas. The reduction occurs in four steps, as written below for one electron equivalent (e^−^ eq) (Rittmann and McCarty, 2001):

(1) $$\begin{array}{l} \begin{array}{c} 1/4CH_{2}O\;+1/2NO_{3}^{-}\to 1/2NO_{2}^{-}+1/4CO_{2}+\;1/4H_{2}O\\ 1/4CH_{2}O\;+\;NO_{2}^{-}+\text{4}H^{+}\to 1/4CO_{2}+\;NO\;+\;3/4H_{2}O\\ 1/4CH_{2}O\;+\;NO\;\to 1/4CO_{2}+\text{1/}2N_{2}O\;+\;1/4H_{2}O\\ 1/4CH_{2}O\;+\text{1/2}N_{2}O\;\to 1/4CO_{2}+\text{1/}2N_{2}+\;1/4H_{2}O \end{array} \end{array}$$

We normalized the above reactions with respect to one mole of dissolved organic carbon (*CH*_2_*O*), so that the coefficients will represent the stoichiometric relationships between reactants and products for a unit mole of consumed organic carbon, as follows:

(2) $$\begin{array}{l} \begin{array}{c} CH_{2}O+2NO_{3}^{-}\to 2NO_{2}^{-}+CO_{2}+H_{2}O\\ CH_{2}O+4NO_{2}^{-}+4H^{+}\to CO_{2}+4NO+3H_{2}O\\ CH_{2}O+4NO\to CO_{2}+2N_{2}O+H_{2}O\\ CH_{2}O+2N_{2}O\to CO_{2}+2N_{2}+H_{2}O \end{array} \end{array}$$

Accurate parameterization of some of the intermediate reactions was difficult because (1) the concentration of N_2_O was very low (i.e., of the order of 10^−6^–10^−3^ mM), indicating its consumption is faster than generation, and (2) no NO measurement was available. Therefore, we simplified the denitrification process into two step reactions by assuming relatively fast dynamics of *NO* and *N*_2_*O*, i.e.,

(3) $$\begin{array}{l} CH_{2}O+2NO_{3}^{-}\to 2NO_{2}^{-}+CO_{2}+H_{2}O \end{array}$$

(4) $$\begin{array}{l} CH_{2}O+4/3NO_{2}^{-}+4/3H^{+}\to 2/3N_{2}+CO_{2}+5/3H_{2}O \end{array}$$

We also consider the synthesis of biomass (*C*_5_*H*_7_*O*_2_*N*) as follows:

(5) $$\begin{array}{l} CH_{2}O+1/5NH_{4}^{+}\to 1/5C_{5}H_{7}O_{2}N+3/5H_{2}O+1/5H^{+} \end{array}$$

Microbes obtain energy through anaerobic respiration pathways, reactions (3) or (4) or both, depending on which electron acceptors ($NO_{3}^{-}$ or $NO_{2}^{-}$) are available in the environment. To account for this coupling, we combined equations (3) and (4) with (5) as follows:

(6) $$\begin{array}{l} CH_{2}O+2f_{1}NO_{3}^{-}+\frac{1}{5}\left(1-f_{1}\right)NH_{4}^{+}\to 2f_{1}NO_{2}^{-}+f_{1}CO_{2}\\ +\frac{1}{5}\left(1-f_{1}\right)C_{5}H_{7}O_{2}N \end{array}$$

(7) $$\begin{array}{l} CH_{2}O+\frac{4}{3}f_{2}NO_{2}^{-}+\frac{1}{5}\left(1-f_{2}\right)NH_{4}^{+}\to \frac{2}{3}f_{2}N_{2}+f_{2}CO_{2}\\ +\frac{1}{5}\left(1-f_{2}\right)C_{5}H_{7}O_{2}N \end{array}$$

where *f*_1_ and *f*_2_ denote the fractions of energy production associated with $NO_{3}^{-}$ and $NO_{2}^{-}$. Note that reactions and share the same electron donor (*CH*_2_*O*), but involve different electron acceptors (i.e., $NO_{3}^{-}$ and $NO_{2}^{-}$), thus representing *alternative* pathways for the production of biomass (*C*_5_*H*_7_*O*_2_*N*). In the above reactions, we excluded *H*^+^ and *H*_2_*O* to focus on carbon and nitrogen balances. For simplicity, hereafter we use *DOC* (dissolved organic carbon), *DIC* (dissolved inorganic carbon) and *BM* (biomass) to denote *CH*_2_*O*, *CO*_2_ and *C*_5_*H*_7_*O*_2_*N*.

### Mass balances of nutrients and biomass

In a homogeneous batch reactor, mass balances of substrates and biomass can be written based on stoichiometric equations (6) and (7) as follows:

(8) $$\frac{d}{dt}\;\left[\begin{array}{c} x_{DOC}\\ x_{NO_{3}^{-}}\\ x_{NO_{2}^{-}}\\ x_{N_{2}}\\ x_{DIC}\\ x_{BM} \end{array}\right]=\left[\begin{array}{cc} -1 & -1\\ -2f_{1} & 0\\ 2f_{1} & -4f_{2}/3\\ 0 & 3f_{2}/2\\ f_{1} & f_{2}\\ (1-f_{1})/5 & (1-f_{2})/5 \end{array}\right]\left[\begin{array}{c} r_{1}\\ r_{2} \end{array}\right]$$

In the above equation, *x* denotes concentrations of reactants and products, and *r*_1_ and *r*_2_ are reaction rates associated with $NO_{3}^{-}$ and $NO_{2}^{-}$, respectively. We modify the mass balances of *BM* and *DOC* to account for biomass degradation and the impact on the formation of organic carbon as follows:

(9) $$\begin{array}{l} \frac{dx_{BM}}{dt}=\frac{1-f_{1}}{5}r_{1}+\frac{1-f_{2}}{5}r_{2}-k_{\deg}x_{BM} \end{array}$$

(10) $$\begin{array}{l} \frac{dx_{DOC}}{dt}=-r_{1}-r_{2}+5k_{\deg}x_{BM} \end{array}$$

where *k*_deg_ denotes the rate of biomass degradation. The coefficient factor “5” on the right hand side of Equation (10) denotes the carbon-based stoichiometric relationship between *BM* (i.e., *C*_5_*H*_7_*O*_2_*N*) degradation and the resulting increase of *DOC* (i.e., *CH*_2_*O*).

### Description of metabolic regulation

It is often that simple Monod-type kinetic equations do not properly describe biogeochemical reactions (Tang and Riley, 2013), which might be ascribed to the lack of description for the regulated metabolic behaviors of microbial communities. Regulation is a hallmark of microbial metabolism, which therefore should be a key component of biogeochemical modeling. Microbes regulate metabolism through the control of enzyme syntheses (and their activities). To account for those regulatory processes, we modeled the *DOC* uptake rate through the reaction *i* (*r*_*i*_) as being regulated by the enzyme concentration (*e*_*i*_), i.e.,

(11) $$\begin{array}{l} r_{i}=e_{i}r_{i}^{kin},\;i=1,2 \end{array}$$

where $r_{i}^{kin}$ denotes the kinetic form of (unregulated) reaction rate. The synthesis of enzyme requires the energy and material resources (i.e., internal resources) such as ATP, NADH, DNA polymerases, RNA polymerases, ribosomes, and so on. Due to their limited availability, microbes allocate resources among different enzymes to selectively activate certain reactions. The cybernetic model provides a rule for this resource allocation based on “the return-on-investment” concept, i.e., how much microbes will gain profit (such as nutrient uptake rates or growth rates) by investing internal resources for the synthesis of certain enzymes.

The RSCM applies the same rule for the resource allocation, but in a more structured way. That is, the model accounts for the resource balance in combination with the two steps of enzymes synthesis: transcription (i.e., the information flow from genes into transcripts) and translation (i.e., from transcripts to enzymes). With a focus on the dynamics of transcripts and enzymes, we treated the genes as part of the resource pool to minimize the number of parameters. The resulting model contains mass balance equations for the three major components associated with regulation: the resource pool (denoted by R), transcripts (T), and enzymes (E). The equations are given as follows:

(12) $$\begin{array}{l} \frac{d\rho_{i}}{dt}=\alpha_{R,i}+u_{i}r_{R,i}-\beta_{R,i}\rho_{i} \end{array}$$

(13) $$\begin{array}{l} \frac{d\tau_{i}}{dt}=r_{T,i}-\beta_{T,i}\tau_{i} \end{array}$$

(14) $$\begin{array}{l} \frac{de_{i}}{dt}=r_{E,i}-\beta_{E,i}e_{i} \end{array}$$

where ρ_*i*_ and τ_*i*_ denote the concentrations of the resource pool and transcripts associated with the reaction *i*; *r*_*R,i*_, *r*_*T,i*_, and *r*_*E,i*_ represent the kinetics of synthesis of resource pools, transcripts and enzymes, respectively; α_*R,i*_ is the constitutive synthesis rate of resource pool *i*; β_*R,i*_, β_*T,i*_, and β_*E,i*_ denote the parameters of degradation rates of resource pool R_*i*_, transcript T_*i*_, and enzyme E_*i*_, respectively. The three terms on the right hand side of Equation (12), respectively, denote the constitutive synthesis rate, inductive synthesis rate, and the degradation rate of resource pool associated with the reaction *i*, where the variable *u*_*i*_ denotes the control of inductive synthesis of resource. Similarly, the two terms on the right hand side of Equations (13) and (14) denote the synthesis and degradation rates of transcripts and enzymes. Note that the resource allocation is determined by the cybernetic variable *u*_*i*_ in Equation (12), which subsequently dictates the syntheses of transcripts and enzymes as in Equations (13) and (14).

The control variable *u*_*i*_ in Equation is determined by the cybernetic control law termed the matching law (Young and Ramkrishna, 2007) as follows:

(15) $$\begin{array}{l} u_{i}=\frac{p_{i}}{\underset{j}{\sum }p_{j}} \end{array}$$

where *p*_*i*_ is the return-on-investment (or profit), which reflects the expected metabolic benefit derived from the allocation of internal resources for the syntheses of transcripts and enzymes and for the subsequent catalysis of specific biogeochemical reactions (see Figure 2). In other words, the variable *p*_*i*_ denotes the contribution that the reaction *i* makes to the postulated metabolic objective function of the microbial community. The typical choice of this objective includes maximization of carbon uptake rate and growth rate; we set *p*_*i*_ to *r*_*i*_ (i.e., carbon uptake rate) in this work. The functional form of *u*_*i*_ in Equation (15) was heuristically determined in earlier cybernetic models, but was later theoretically derived from an optimal control theory (Young and Ramkrishna, 2007).

The typical cybernetic modeling formulation adds the post-translational control of enzyme activities as well, which was not accounted for in the current form of RSCM due to its relatively low impact on the prediction as discussed in our previous denitrification modeling analysis (Song and Liu, 2015). However, the enzyme activity control is an important regulatory mechanism in general (Oliveira and Sauer, 2012) and may need to be incorporated into the model in certain circumstances, e.g., in the case that the system exhibits fast nonlinear regulatory dynamics over a short time period, because it enables the prompt control of metabolic shifts through allosteric hindrance of enzyme activities.

### Kinetics

We used Monod-type equations to describe the unregulated kinetic rates (i.e., $r_{i}^{kin}$) of ${\text{NO}}_{3}^{-}$ and ${\text{NO}}_{2}^{-}$ in Equation (11), i.e.,

(16) $$\begin{array}{l} r_{1}^{kin}=k_{1}\frac{x_{DOC}}{K_{d,1}+x_{DOC}}\frac{x_{NO_{3}^{-}}}{K_{a,1}+x_{NO_{3}^{-}}}\\ r_{2}^{kin}=k_{2}\frac{x_{DOC}}{K_{d,2}+x_{DOC}}\frac{x_{NO_{2}^{-}}}{K_{a,2}+x_{NO_{2}^{-}}} \end{array}$$

A similar form of kinetic equations was considered for describing the synthesis rates of resources, transcripts, and enzymes, i.e.,

(17) $$\begin{array}{l} r_{R,i}=\{\begin{array}{c} k_{R,1}\frac{x_{DOC}}{K_{d,1}+x_{DOC}}\frac{x_{NO_{3}^{-}}}{K_{a,1}+x_{NO_{3}^{-}}},i=1\\ k_{R,2}\frac{x_{DOC}}{K_{d,2}+x_{DOC}}\frac{x_{NO_{2}^{-}}}{K_{a,2}+x_{NO_{2}^{-}}},i=2 \end{array}\\ r_{T,i}=k_{T,i}\frac{\rho_{i}}{K_{T,i}+\rho_{i}}\\ r_{E,i}=k_{E,i}\frac{\tau_{i}}{K_{E,i}^{T}+\tau_{i}}\frac{\rho_{i}}{K_{E,i}^{R}+\rho_{i}} \end{array}$$

where the *k*'S and *K*'s denote reaction rate and half-saturation constants associated with specific reactions as implied by their subscripts. It is important to note that (1) any alternative forms of kinetic models can be used in the cybernetic modeling formulation, and (2) even in the case where simple Monod-type kinetics were used for $r_{i}^{kin}$ (as above), the actual kinetics in Equation (11) show much more complex dynamics due to the regulatory processes represented in Equations (12–15).

### Parameter identification

We determined key parameters of the denitrification model through the data fit such that the sum of the squared errors between simulations and measured data is minimized. Experimental data used for this purpose included temporal profiles of substrates (${\text{NO}}_{3}^{-}$, ${\text{NO}}_{2}^{-}$, DOC, and DIC) and enzymes (NarG, NapA, NosZ1, and NosZ2). Specifically, the catalysis of the reduction from ${\text{NO}}_{3}^{-}$ to ${\text{NO}}_{2}^{-}$ in Equation (6) was parameterized in association with the dynamics of NarG/NapA enzyme concentrations. The reaction from ${\text{NO}}_{2}^{-}$ to N_2_ is originally composed of three reduction steps catalyzed by NirS/NirK, Nor, and NosZ1/NosZ2, respectively (Figure 1B), but as mentioned earlier, we associated it only with the dynamics of NosZ1/NosZ2 enzymes that catalyze the last step of reduction, due to the lack of other enzyme measurements. The fitted parameters include the rate constants of carbon uptake (*k*_1_, *k*_2_), the rate constants of the syntheses of internal resources (*k*_*R*,1_, *k*_*R*,2_), transcripts (*k*_*T*,1_, *k*_*T*,2_), and enzymes (*k*_*E*,1_, *k*_*E*,2_), the biomass degradation rate (*k*_deg_), and the fraction of energy production associated with $NO_{3}^{-}$ reduction to $NO_{2}^{-}$ (*f*_1_). Due to the limited availability of experimental data, the parameters associated with the dynamics of internal resources and transcripts (*k*_*R*,1_, *k*_*R*,2_, *k*_*T*,1_, and *k*_*T*,2_) were indirectly identified by fitting the profiles of nutrients and enzymes. Other parameters were fixed based on the literature values (i.e., half saturation constants) or determined by manual adjustment (*f*_2_, α 's, and β 's). For fitted parameters, we used the bootstrapping technique to quantify the uncertainty in the estimates based on 95% confidence intervals. Bootstrapping was performed by estimating the best-fit parameter values for 500 experimental data sets, where each data set was created by randomly sampling a uniform distribution of the measured values at each time point. The sampled values varied within ±standard deviation from the mean experimental value. The resulting parameter sets containing values below the 2.5th and above the 97.5th quantile for at least one parameter were removed. The lowest and highest values of each parameter in the remaining parameter sets were taken as the lower and upper 95% confidence limits, respectively. The resulting parameter values (with lower and upper bounds) are summarized in Table 1. The initial concentrations of all variables including nutrients, internal resources, transcripts, and enzymes are shown in Table 2.

**Table 1:** Model parameter values used for the simulation of the two-step denitrification process.

**Parameter**	**$i=1\left(NO_{3}^{-}\to NO_{2}^{-}\right)$**	**$i=2\left(NO_{2}^{-}\to N_{2}\right)$**	**Source for parameter value**
*f*_*i*_[-]	0.56 [0.33, 0.69]	0.99	Data fit
*k*_*i*_[^*^]	6.23 [5.25, 11.0]	5.71 [4.96, 6.08]	Data fit
*K*_*d,i*_[mM]	0.25	0.25	Yan et al., 2016^**^
*K*_*a,i*_[mM]	0.001	0.004	Rittmann and McCarty, 2001
*k*_deg_[^*^]	0.11 [0.05, 0.30]		Data fit
α_*R,i*_[1/d]	0.2	0.2	Song and Liu, 2015^**^
β_*R,i*_[1/d]	0.8	0.8	Assumed β_*R,i*_ = β_*E,i*_
β_*T,i*_[1/d]	0.8	0.8	Assumed β_*T,i*_ = β_*E,i*_
β_*E,i*_[1/d]	0.8	0.8	Song and Liu, 2015^**^
*k*_*R,i*_[1/d]	2.84 [2.83, 18.1]	1.03 [0.68, 2.59]	Data fit
*k*_*T,i*_[1/d]	0.13 [0.06, 0.20]	0.25 [0.08, 0.31]	Data fit
*k*_*E,i*_[1/d]	1.84 [1.27, 2.69]	1.56 [1.45, 3.43]	Data fit
*K*_*T,i*_[-]	0.25	0.25	Assumed *K*_*T,i*_ = *K*_*d,i*_
*K*_*E,i*_[pmol/g soil]	0.25	0.25	Assumed *K*_*E,i*_ = *K*_*d,i*_

*The values in brackets denote the lower and upper limits of 95% confidence intervals for fitted parameters*.

* *Units: k_i_[=] mM·(g soil/pmol enzyme)/d; k_deg_[=] [mmol/mmol BM/d]*.

** *Fixed with comparable values used in the corresponding literature*.

**Table 2:** Initial condition used for the simulation of denitrification dynamics: *DOC* = dissolved organic carbon, *DIC* = dissolved inorganic carbon, and *BM* = biomass; ρ, τ and *e* denote the concentrations of internal resource, transcript and enzyme, respectively; the subscripts 1 and 2 denote the association with $NO_{3}^{-}$ and $NO_{2}^{-}$ reduction pathways, respectively.

**Nutrient**	**Concentration**	**Regulatory molecules**		**Concentration**
*DOC*[mM]	61.1^*^	Internal resource [–]	ρ_1_	0.001
$NO_{3}^{-}$[mM]	18.3^*^		ρ_2_	0.001
$NO_{2}^{-}$[mM]	0^*^	Transcript [–]	τ_1_	0.001
*N*_2_[mM]	0		τ_2_	0.001
*DIC*[mM]	1	Enzyme [pmol/g soil]	*e*_1_	0.001
*BM*[mM]	1.5		*e*_2_	0.001

* *Initial concentrations of DOC, ${\text{NO}}_{3}^{-}$, and ${\text{NO}}_{2}^{-}$ measured by Li et al. (2017). All other initial concentrations denote assumed values*.

## Results

### Fitting results and prediction of time lags in enzyme response

**Figure 3:**
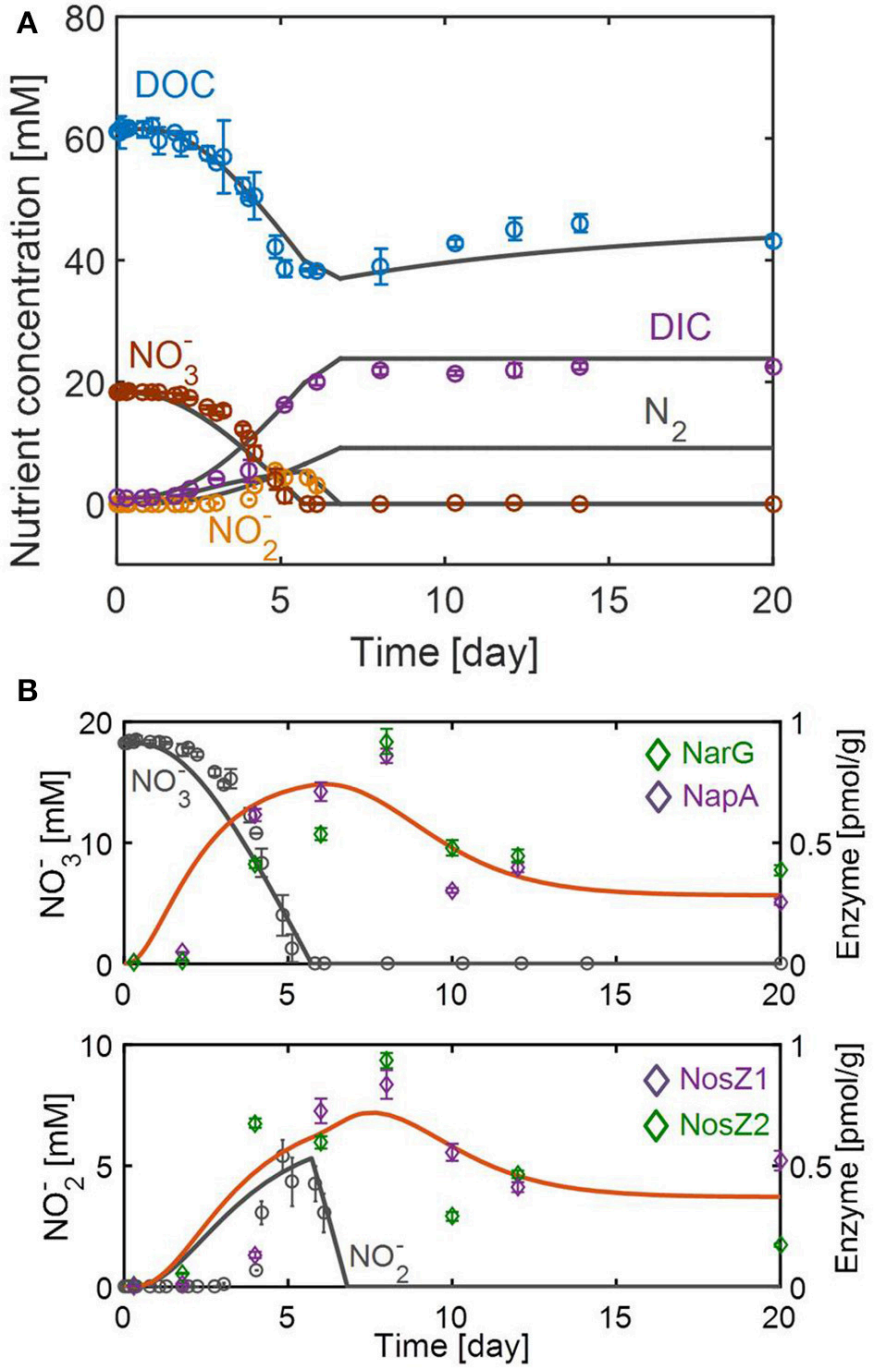
Model fits and predictions of the dynamic changes in **(A)** substrates and **(B)** enzymes associated with ${\text{NO}}_{3}^{-}$ reduction to ${\text{NO}}_{2}^{-}$ (top) and the subsequent ${\text{NO}}_{2}^{-}$ reduction to N_2_ (bottom). Symbols in **(A,B)** represent the average concentrations of nutrients (DOC, DIC, ${\text{NO}}_{3}^{-}$, and ${\text{NO}}_{2}^{-}$) and enzymes (NarG, NapA, NosZ1, and NosZ2) obtained from three and two biological replicates, respectively. Error bars denote the standard deviations of each measurement.

The batch denitrification experiment by Li et al. (2017) with only one initial condition (Table 2) did not provide sufficient data for the accurate determination of all parameters in the RSCM. Thus, we fixed some of the parameters that might not be directly determinable from simple batch data, including half-saturation constants and the parameters associated with constitutive synthesis and degradation of regulatory molecules. This strategy led us to reproducibly determine the remaining parameters through optimal data fit (see Table 1 and Figure 3).

The resulting model successfully fitted the overall dynamics of nutrients (Figure 3A) and enzymes (Figure 3B). The model also captured the non-intuitive trend of DOC profile, i.e., the gradual increase of DOC after day 6 (when no further denitrification was actively taking place), by attributing it to biomass degradation (see Equation 10). The model also predicted N_2_ production on the assumption that all ${\text{NO}}_{2}^{-}$ is converted into N_2_ through the stoichiometric relation given in Equation (7). Figure 3B shows that enzyme responses to the change of ${\text{NO}}_{3}^{-}$ and ${\text{NO}}_{2}^{-}$ were significantly delayed. Data on the top panel of Figure 3B shows that the ${\text{NO}}_{3}^{-}$ reduction rate reached its maximal value around day 4, while the maximal concentrations of catalyzing enzymes (i.e., NarG and NapA) occurred about 4 days later. The RSCM simulated the maximal reduction rate and enzyme concentrations to occur slightly earlier, but its prediction of the time delay in enzyme expression was aligned with experimental observation. The delayed enzymatic response was also inferred for ${\text{NO}}_{2}^{-}$ reduction from the observation that the maximal concentrations of NosZ1 and NosZ2 enzymes appeared about 1 day after the depletion of ${\text{NO}}_{2}^{-}$. However, it was difficult to accurately estimate the time delay in this case because of (1) the uncertainty about the exact time when the ${\text{NO}}_{2}^{-}$ reduction rate reached its maximum (due to the non-monotonic profile shape), and (2) the absence of the measurement of enzymes other than NosZ1 and NosZ2, which catalyze the last step of denitrification. Continued synthesis of enzymes even after substrate depletion is an interesting phenomenon, which we may describe as *dynamic momentum in metabolic control*. In addition to delayed enzymatic responses, the data showed that enzyme concentrations (for both ${\text{NO}}_{3}^{-}$ and ${\text{NO}}_{2}^{-}$ reduction reactions) were maintained from day 10 up to day 20. The RSCM predicted this persistence of enzyme levels as the result of dynamic balance between constitutive enzyme synthesis and degradation rates, as in Equations (12–14).

### Dynamic control of denitrification reactions

**Figure 4:**
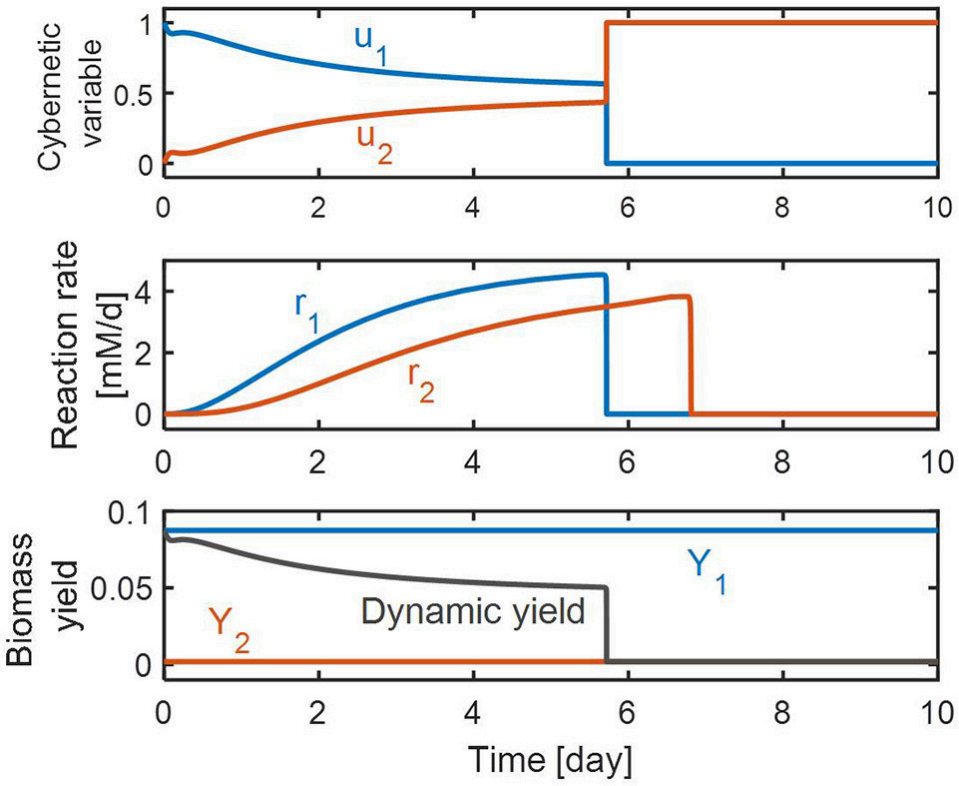
Model predictions of the dynamic changes in cybernetic variables **(top)**, reaction rates **(middle)**, and biomass yield **(bottom)**. In the bottom panel, *Y*_1_ and *Y*_2_ denote the stoichiometric biomass yields associated with ${\text{NO}}_{3}^{-}$ and ${\text{NO}}_{2}^{-}$ reductions, respectively.

Figure 4 shows the effects of control realized by the cybernetic variables on the ${\text{NO}}_{3}^{-}$ and ${\text{NO}}_{2}^{-}$ reduction rates and the biomass yield. On the top panel, the value of *u*_1_ is initially high, and gradually decreases. This means that the initial allocation of the resource pool is directed primarily toward promoting ${\text{NO}}_{3}^{-}$ reduction; its portion decreases as the amount of ${\text{NO}}_{2}^{-}$ increases as implied by the increasing value of *u*_2_. The middle panel shows the rates of ${\text{NO}}_{3}^{-}$ and ${\text{NO}}_{2}^{-}$ reduction as their concentrations change with time. The RSCM predicted these two competitive reactions can take place *simultaneously* until ${\text{NO}}_{3}^{-}$ is depleted. This is an interesting finding from a modeling point of view because the conventional cybernetic control that considers the control of the inductive enzyme synthesis directly in the enzyme balance equation usually predicts competitive reactions (such as consecutive ${\text{NO}}_{3}^{-}$ and ${\text{NO}}_{2}^{-}$ reduction reactions) to occur in a *sequential* manner as observed in our previous denitrification model. Prediction of the simultaneous occurrence of competitive reactions by the conventional cybernetic model often requires the consideration of additional reactions, which is not necessary for the RSCM that *indirectly* accounts for the control of inductive enzyme synthesis through the allocation of internal resources. On the bottom panel, we showed the prediction for dynamic changes in the community's biomass “yield” during denitrification. The biomass yield at each time instant was calculated as the ratio between the biomass production rate and the DOC (or carbon) uptake rate, i.e.,$\overset{2}{\underset{i=1}{\sum }}Y_{i}r_{i}/\overset{2}{\underset{i=1}{\sum }}r_{i}$. That is, the biomass yield is a dimensionless quantity representing the amount of produced biomass [mM] per the unit amount of consumed DOC [mM]. The biomass synthesis initially is high due to the higher contribution of ${\text{NO}}_{3}^{-}$ reduction (because *f*_1_ < *f*_2_; see section Methods); gradually decreases for the first 3 days; maintains an almost constant level until day 5.7 where the relative ratio of ${\text{NO}}_{3}^{-}$ and ${\text{NO}}_{2}^{-}$ reductions does not change much (as shown by the flattened profiles of *u*_1_ and *u*_2_. The ${\text{NO}}_{2}^{-}$ reduction continues until day 6.8 while the biomass yield becomes very small after day 5.7, which is because the high value of *f*_2_ is close to 1 (i.e., 0.99) in Equation (7).

### Transcripts playing a role as a dynamic control point

**Figure 5:**
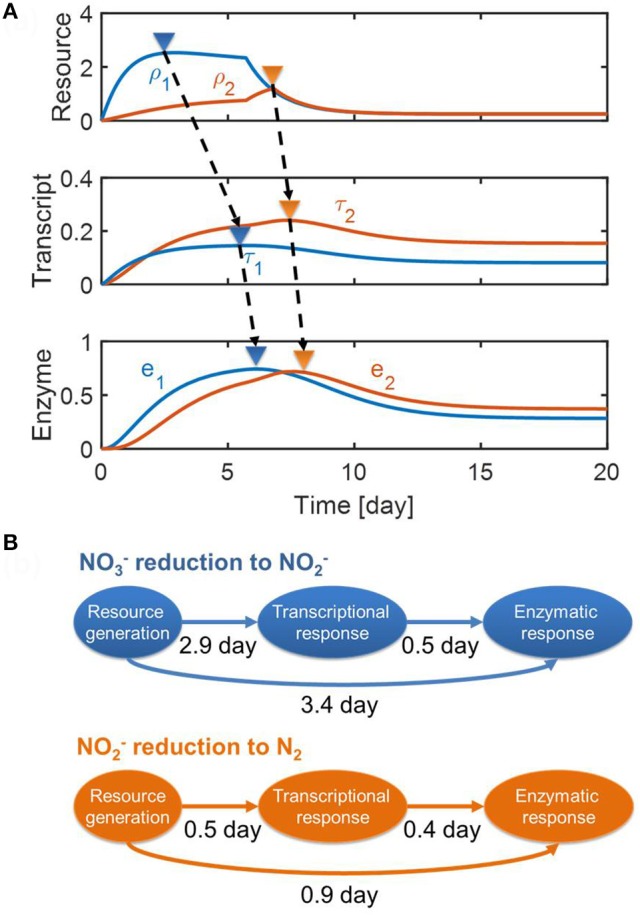
Model predictions of **(A)** the dynamic changes in resources, transcripts and enzymes and **(B)** time delays from resource generation to transcriptional and enzymatic responses. In **(A)**, the upside-down triangles denote the time where each variable reaches its maximal value.

In Figure 5, we provided (a) the dynamic profiles of resources, transcripts, and enzymes, and (b) time lags quantified based on the differences between the peak times of each variable (as marked by upside-down triangles in Figure 5A). For the reduction of ${\text{NO}}_{3}^{-}$ to ${\text{NO}}_{2}^{-}$, the figure shows that a long lag in enzyme response is fundamentally caused by the interplay of enzymes with transcripts and internal resources, which do not quickly decay even after the substrates are depleted. The same interpretation also applies to the conversion of ${\text{NO}}_{2}^{-}$ to N_2_, but the level of time delay is less pronounced, particularly when considering the fact that the ${\text{NO}}_{2}^{-}$ reduction was associated with the dynamics of NosZ1/NosZ2 enzymes that catalyze the last step of reduction (see section Methods). This result—i.e., the prediction of a longer time delay in enzymatic response for the ${\text{NO}}_{3}^{-}$ reduction (i.e., 3.4 day) in comparison to the ${\text{NO}}_{2}^{-}$ reduction (0.9 day)—agrees with experimental observation, as shown in Figure 3.

For the reduction of ${\text{NO}}_{3}^{-}$ to ${\text{NO}}_{2}^{-}$, the model predicted a long delay between the resource generation and the transcriptional response (i.e., about 3 days), while predicting a relatively short delay between transcriptional and enzymatic responses (0.5 day) (top panel of Figure 5B). This indicates that the transcriptional response might be a key control point in ${\text{NO}}_{3}^{-}$ reduction. However, this was not the case for the ${\text{NO}}_{2}^{-}$ reduction where time delays between three processes were comparable (i.e., 0.5 and 0.4 days, respectively) (bottom panel of Figure 5B). Interestingly, time delays between transcriptional and enzymatic responses were predicted to be similar for ${\text{NO}}_{3}^{-}$ (0.5 day) and ${\text{NO}}_{2}^{-}$ reduction (0.4 day).

This result also reveals the complex interplay between resources, transcripts, and enzymes in denitrification. The dynamic profiles of enzymes (bottom panel of Figure 5A) bear a qualitative resemblance to the profiles of reaction rates shown on the middle panel of Figure 4. That is, profiles in both figures show that initially ${\text{NO}}_{3}^{-}$ reduction is dominant but is subsequently exceeded by ${\text{NO}}_{2}^{-}$ reduction when ${\text{NO}}_{3}^{-}$ is depleted. The initial trend was also the same for resource abundance profiles (top panel of Figure 5A). In contrast, the transcript abundance profiles (middle panel of Figure 5A) are fundamentally different; the abundance of transcripts associated with ${\text{NO}}_{3}^{-}$ reduction is relatively low throughout the entire simulation, despite being at similar abundance to ${\text{NO}}_{2}^{-}$ reduction transcripts initially. Prediction of these distinct features of transcripts is an outcome of data fit in order to match the overall denitrification dynamics where ${\text{NO}}_{3}^{-}$ reduction is more dominant than ${\text{NO}}_{2}^{-}$ reduction, while the corresponding enzyme concentrations that catalyze them are comparable. This implies that transcripts might play a role of controlling the overall dynamics of regulation at the interface between resources and enzymes.

### Highly correlated dynamics of transcripts and enzymes

**Figure 6:**
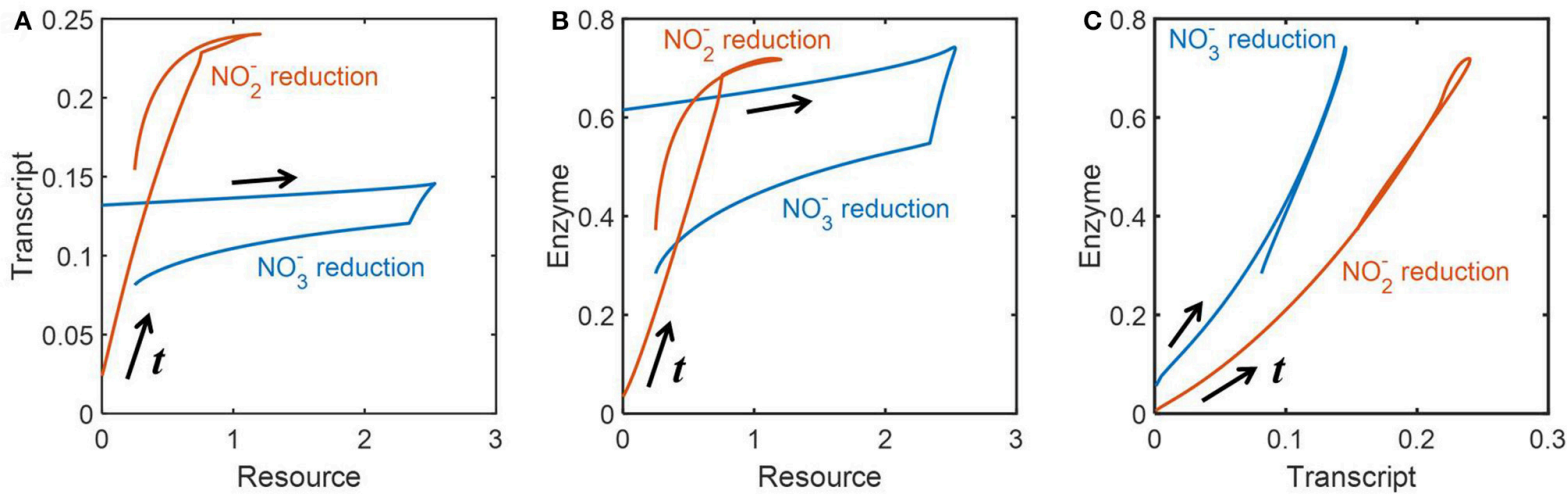
Phase diagrams of resources, transcripts and enzymes: the dynamic relationships **(A)** between the concentrations of resources and transcripts, **(B)** between the concentrations of resources and enzymes, and **(C)** between the concentrations of transcripts and enzymes. The time scales of all data are adjusted to remove the effect of time delay based on the quantification in Figure 5B.

We also analyzed how the abundances of resources, transcripts and enzymes were dynamically inter-related. To remove the effect of time delay, we appropriately adjusted the time scales of the profiles of these three variables to draw phase diagrams between all possible pairs of three variables. Figures 6A,B provide the relationships between resources and transcripts, and between resources and enzymes, respectively; we found the reasonably close relationships between those pairs for ${\text{NO}}_{2}^{-}$ reduction, while there was no such correlation for ${\text{NO}}_{3}^{-}$ reduction, i.e., transcript and enzyme levels did not proportionally change to the abundance of resources. In Figure 6C, we consistently observed, however, the close relationships between transcripts and enzymes for both ${\text{NO}}_{3}^{-}$ and ${\text{NO}}_{2}^{-}$ reduction; surprisingly, their relationships were almost linear along both increasing and decreasing phases.

## Discussion

### Key features of the RSCM

We provided a new conceptual platform (termed RSCM) for modeling complex microbial communities. There are several unique features of the RSCM as highlighted in the following. First, the RSCM is built upon the functional enzyme-based approach, which enables the consistent model formulation of complex microbial communities. This enzyme-based parameterization is particularly useful in circumstances where metabolic functions of individual organisms are difficult to uniquely characterize (and therefore difficult to group). Second, the RSCM elaborates regulatory control by accounting for transcription and translation, the products of which can be quantified. Considering the difficulty in obtaining reliable estimates of guild-specific biomass for the function guild-based approach, the ability to directly quantify transcripts and enzyme concentrations is likely an advantage of the enzyme-based approach. As another key distinction, the RSCM describes the dynamic microbial regulation based on the cybernetic approach. In the current practices of biogeochemical modeling, metabolic regulation is often accounted for based on empirical inhibition kinetics, which not only increases the number of parameters to identify, but also may lead to less accurate predictions (Tang and Riley, 2013). In contrast, the cybernetic model can provide reliable predictions over a wider range of conditions with a fewer number of parameters as demonstrated in the past case studies of metabolic modeling (Ramkrishna and Song, 2012; Song et al., 2013a). Data requirement for parameter identification is accordingly lesser for the cybernetic model due to the absence of inhibition-associated parameters, which was necessary for empirical formulation. Based on the principle of Occam's razor (Gauch, 2003), the simplest model is preferred over over-parameterized models, if performance is the same. Accounting for metabolic control based on the cybernetic approach holds a significant advantage in modeling environmental biogeochemical processes, not only that experimental data required for parameter identification is usually insufficient, but also that *a priori* knowledge is unavailable for inhibition control in a complex system.

### Insights into metabolic lags and the role of transcripts

The RSCM showed that enzymatic time lags observed in denitrification experiments can be caused by the complex interplay among internal resource molecules, transcripts and proteins. The delayed enzymatic response could be ascribed to microbes' regulatory machinery that controls energetically-expensive enzyme synthesis to ensure its survival in frequently varying environment (Ramkrishna et al., 1987). This postulate is rational because immediate and frequent shifts in enzyme settings in response to environmental variation can lead to the rapid depletion of energy required for microbial growth and maintenance due to the cost for protein synthesis (Wessely et al., 2011; Noor et al., 2016).

The RSCM also provided insights in regard to the potential role of transcripts in regulatory dynamics of denitrifying microbial communities. First, it suggests that the overall time delay in denitrification may be primarily controlled by the transcriptional response. Model predictions showed that transcriptional response can be slower than or comparable to enzymatic responses depending on the type of reactions. This implies that accurate simulation of denitrification requires an appropriate consideration of transcriptional dynamics in modeling. Second, the abundances of transcripts may not necessarily reflect the level of reaction activities. The simulation showed that the transcripts associated with ${\text{NO}}_{3}^{-}$ reduction were less abundant than those associated with ${\text{NO}}_{2}^{-}$ reduction even though the former reaction was dominant until the depletion of ${\text{NO}}_{3}^{-}$. This suggests that within a given reaction, changes in transcript abundances (e.g., through time) may indicate changes in the rate of that reaction, but that such a comparison cannot be made across reactions. In this regard, it would be beneficial to consider collecting additional complementary data (e.g., gene abundances, metabolomic profiles, etc.) and/or performing advanced tracer experiments using isotopically labeled substrates. Third, the model suggests that transcripts could be used as a proxy for enzymes due to their highly inter-correlated dynamics. This finding is of practical importance considering the relatively higher cost and technical difficulties in directly measuring enzyme concentrations from environmental samples. While we were able to use quantitative enzyme data thanks to the advanced analytical method recently developed by Li et al. (2017), this method is costly and labor intensive in relative to transcript quantification.

### Concluding remarks and future considerations

Using the RSCM framework, we explored how the dynamic interplay between internal resources, transcripts, and enzymes can be considered as a potential mechanism responsible for the non-intuitive biogeochemical dynamics observed in our denitrification experiments. The good agreement we obtained between data and the model indicated that the proposed mechanism is plausible, while we could not provide a direct experimental proof. Further experimental studies would be required for a more conclusive understanding, particularly on the role of transcripts as a control point of regulatory dynamics. One could consider other biological or physical processes that may potentially cause our observed phenomena. For example, the slow process of transcription and translation could be ascribed to nutrient transport across the cell membrane mediated by the membrane-localized proteins (permeases) (Stephanopoulos et al., 1998). As another example, enzyme adsorption to minerals could be a potential process explaining their persistent activities in the sediments (Zimmerman and Ahn, 2010). Despite these caveats, the predictions of RSCM provide fresh insights and novel hypotheses that cannot be derived by simple structured biogeochemical models. Thus, the simulation results provide a reasonable basis for a deeper understanding of the role of microbes in regulating biogeochemical functions. In future applications, the RSCM will be further tested under various other environmental conditions where the importance of accounting for cellular regulation becomes more pronounced, including the transition from oxic to anoxic conditions. We expect the RSCM to serve not only as an independent simulator of denitrification dynamics, but also as a key biogeochemical modeling component of multi-scale reactive-transport models. As will be reported in a near future, the RSCM is currently incorporated into multi-scale simulation software, PFLOTRAN (Lichtner et al., 2015) to investigate hydro-biogeochemical processes and to predict the long-term impact of dam operation on thermo-hydro-biogeochemical dynamics.

## Author contributions

All authors contributed to the design of the research. HS developed the modeling concept and performed simulations. DT, XS, and XC contributed to the parameter identification. HS and DT drafted out the article, which was edited by JS and JF. All authors read and approved the final manuscript.

### Conflict of interest statement

The authors declare that the research was conducted in the absence of any commercial or financial relationships that could be construed as a potential conflict of interest.
